# Optimization of LED Lighting for Clinical Settings

**DOI:** 10.1155/2019/5016013

**Published:** 2019-08-27

**Authors:** Snjezana Soltic, Andrew Chalmers

**Affiliations:** ^1^Professional Engineering, Manukau Institute of Technology, Manukau 2241, New Zealand; ^2^Institute of Biomedical Technologies, Auckland University of Technology, Auckland 1142, New Zealand

## Abstract

The advent of the LED light source has promoted the concept of human-centric lighting (HCL). The LED has also been responsible for increases in the electrical efficiency of lighting systems, coupled with recent improvements in their colour properties. We have found that it is also possible to create a lit environment with enhanced clinical attributes by providing a source spectrum that meets the requirements of the Cyanosis Observation Index (COI). This paper describes the use of a differential evolution (DE) algorithm for the spectral design of a mixed LED light source capable of meeting COI recommendations as well as HCL performance criteria.

## 1. Introduction

Researchers worldwide have for several years been attempting to quantify the benefits of human-centric lighting (HCL). A report for the European Commission has identified a range of potential human-health issues arising from LED lighting [1] (and, one may add, its misapplication). HCL attempts to avoid the pitfalls by the creation of a lit environment that emulates natural lighting and promotes the human circadian response. The relationship between light and the levels of wellbeing of the users was expanded when a third photoreceptor, in addition to rods and cones, was identified in the human eye, early in the new century. These additional light-sensitive photoreceptors in the retinal ganglion cell layer (pRGCs) are directly sensitive to light and primarily responsible for mediating these responses [2–4]. These cells are most sensitive to short-wavelength light with a peak sensitivity in the visible blue light range (446–477 nm) [5].

With that discovery, the effects on circadian rhythms (i.e., mental, physical, and biological changes) could be correlated to specific lighting conditions—in particular, the properties of the light spectrum as well as the absolute level of the lighting. A number of distinct purposes for HCL are now emerging, of which three are readily discerned: biologically effective lighting to improve cognitive performance; emotionally effective lighting to create stimulating environments; and clinically effective lighting to improve health care and treatment facilities in hospitals, clinics, and retirement homes. LED lighting appears to be the driving force for new research into HCL and its applications in a wide range of appropriate settings. LEDs enable control of the lighting, with the prospect of independently varying intensity, spectrum, and colour.

As stated in [6], “what we are really defining here is the ability to influence human experience, physiology, and/or behaviour through the delivery of carefully chosen light qualities with control.” In our approach, HCL harnesses the optimization of the light-source spectrum to achieve maximum comfort and efficiency for the occupants. In the clinical context, this requires good colour rendition in general, as well as the facility for effective observation of patients by clinic staff.

Cyanosis (or, perhaps more correctly, its absence) is regarded by clinicians as an important indicator of a patient's health. It refers to a blue coloration of skin and lips and results from poor oxygenation of the blood which can be an indicator for a range of conditions [7–9]. The COI has been developed as a lighting design objective that enhances the clinician's ability to discern differences between blue and red colour casts of the skin. The cyanosis observation criteria set by the ANZS standard for hospital lighting [10] are 3300 ≤ CCT ≤ 5300 K, plus the COI < 3.3.

The following lighting design objectives flow from these concepts: (i) variable CCT (correlated colour temperature) to stimulate the circadian response; (ii) the best achievable COI and colour rendition properties at each CCT; (iii) the highest achievable luminous efficacy (for best system efficiency) consistent with objectives (i) and (ii).

The purpose of this project is to derive the SPDs (spectral power distributions) of LED mixtures that are capable of providing optimum cyanosis observation, along with excellent colour rendition properties, as well as the highest achievable luminous efficacy of radiation. Since these three objectives impose their own unique demands on the distribution of spectral power, this requires a balancing (and optimization) of their contravariant effects.

## 2. Lighting Background

### 2.1. Correlated Colour Temperature

Correlated colour temperature (CCT) refers to the colour appearance of the source, viewed directly. It is expressed on a numerical scale that represents the temperature of a Planckian radiator having the colour that matches (as nearly as possible) the colour of the test source [11]. In contradistinction to the CCT value, the ambience created by different source colours is described as “warm” for lower CCTs and “cool” for the higher CCTs. Source CCTs between 3300 K and 5300 K have been found to be best for COI purposes [7]. In general lighting practice, it is usual to specify CCTs between 2700 K (approximately the colour of a tungsten filament lamp) and 6500 K (matching noon daylight on a clear summer day). We also allow for the possibility of increasing the CCT to 7500 K to emulate sky light during the daytime hours.

### 2.2. Luminous Efficacy

Luminous efficacy (LE) refers to the capacity of the source to produce visible light output efficiently and is measured in lumens per watt. The choice to work with LEDs was influenced by their generally high performance in this context. In the simulations reported here, we quote the LER (luminous efficacy of the radiation) of each LED mixture, which needs to be as high as possible to provide a modern, energy-efficient lighting design.

The LER is defined in equation (1), and the overall luminous efficacy (LE) in equation (2):

(1) $$\begin{array}{c} \text{LER}=\frac{K_{\text{m}}\int_{\lambda }V\left(\lambda \right)S\left(\lambda \right)d\lambda }{\int S\left(\lambda \right)d\lambda }, \end{array}$$

(2) $$\begin{array}{c} \text{LE}=\;\frac{K_{\text{m}}\int_{\lambda }V\left(\lambda \right)S\left(\lambda \right)d\lambda }{P_{E}}. \end{array}$$

*K*_m_ is the maximum luminous efficacy of radiation (≈683 lumen per watt), *S*(*λ*) is the spectral distribution of the light source, and *V*(*λ*) is the CIE spectral sensitivity function for human photopic vision. Hence, the LER assesses the “lighting content” of the spectrum by comparing the visible light output (in lumens) against the total radiant output (in watts). LE, on the contrary, is based on the electrical power consumption *P*_E_ which needs to supply the conversion losses in the light source as well as the total radiant output, as shown in the following equation:

(3) $$\begin{array}{c} P_{\text{E}}=\text{CL}+\int S\left(\lambda \right)d\lambda , \end{array}$$

where CL represents the conversion losses which depend on the physical processes in the lamp.

It is clear that the denominator in equation (2) is always greater than that in equation (1), and so LE is always less than LER. However, the LER is simpler to predict since it depends solely on the spectrum of the source.

### 2.3. Colour Rendition

This refers to the ability of the source to illuminate surfaces such that their colours appear as natural as possible [12]. The current internationally agreed method for the classification of the colour rendition properties of a source is the CIE colour rendering index (CRI), also known as *R*_a_ [13]. For exacting applications such as this study, we have supplemented this with the IESNA's TM-30-15 method for the specification of colour fidelity *R*_f_ [14]; and we quote both *R*_a_ and *R*_f_ (and several subsidiary indices) in our simulations. Both systems employ sets of test colours which are illuminated in turn by test and reference source spectra. (This is a conceptual comparison which is carried out using numerical calculations.) The reference spectrum is always selected to have the same CCT as the test spectrum. The colour difference for each sample, as viewed under the two sources, is calculated numerically, and the results are manipulated and averaged to provide an index on a scale having a maximum of 100 (which would mean perfect colour conformance between the test and reference sources). In practice, the aim is to achieve the highest possible *R*_a_ and *R*_f_ at a given CCT, and we do not recommend spectral designs having *R*_a_ or *R*_f_ values below 83.

### 2.4. Clinical Lighting Practice

The lighting of hospitals and other healthcare facilities is guided by the standards or codes of practice adopted for use in different countries. These are selected as the main focus for this discussion since they represent the distilled wisdom of groups of expert practitioners in the countries concerned.

The major concerns of such documents are the levels of lighting (e.g., lux levels or illuminances) as well as glare control of the light sources. While these properties are naturally of great importance, they are not matters of concern in the present paper since our focus is on the colour and colour-rendition properties of the light sources.

In this context, the majority of hospital and clinical lighting has up to now been based on the use of tubular fluorescent (and, in some cases, compact fluorescent) lamps. LED lighting has only recently come into consideration, and the concept of using LEDs to achieve HCL is a very recent development (and will take time to become reflected in codes and standards).

Colour and colour rendition are important properties of any light source, and recommendations on these are normally provided in codes and standards, as summarized in Table 1.

It is noteworthy that the American recommendations are largely descriptive and are less prescriptive than the others in Table 1. However, all the listed recommendations are seen to be in general accord with one another and differ only in particular details.

Various authors have commented on the desirable colour properties for hospital lighting. Alzubaidi and Soori [18] describe the need for a quality-lighting environment, calling for the CRI (*R*_a_) to be ≥80 in all areas of hospitals. Leccese et al. writing in Italy [19] also quote this figure, as well as *R*_a_ ≥ 90 for examination and treatment, preop and recovery rooms, operating theatres, and colour inspection areas. Thorn Lighting [20] also refers to these values for *R*_a_ and adds that the most common CCT used in UK healthcare spaces is 4000 K, while 2700 K may be used where it is desired to provide a “more homely feel.” Mehrotra et al. [21], describing hospital lighting in India, also call for a minimum *R*_a_ of 80 (in general) or 90 (for examination and treatment, etc.).

Similar to the AS/NZS requirement for COI lighting, but following different paths, Bartczak et al. [22] have devised an LED-based tunable illuminator that provides improved contrast between skin and veins as compared to daylight, while Litorja and Ecker [23] have designed a 3-wavelength LED lamp that can enhance visual contrast when viewing veins on the back of human hands.

We note that our proposals will be taking hospital lighting design into new directions. Our work aims to meet, as far as possible, the intentions expressed in Table 1, but with the additional target of meeting the requirements for COI wherever possible and also to provide the capability for circadian-based HCL.

We have aimed to meet or exceed the following numerical indicators:

 
*R*_a_ ≥ 80 (CIE 13.3)
 
*R*_f_ ≥ 80; *R*_fskin_ ≥ 90 (IES TM-30-15)
  COI ≤ 3.3 with 3300 K ≤ CCT ≤ 5300 K (AS/NZS 1680.2.5 : 2018)
  2700 K ≤ CCT ≤ 7500 K (range for HCL).

The intention is to satisfy the *R*_a_, *R*_f_, and *R*_fskin_ requirements at all times and to comply with COI conditions at all times except when extreme CCT values are called for by the needs of HCL. We argue that a requirement for *R*_a_ ≥ 90 (as for examination and treatment areas) can be satisfied by a combination of {*R*_a_ ≥ 80, *R*_f_ ≥ 80, and *R*_fskin_ ≥ 90} since it is the colour of human skin that is most critical in such situations. A value over 90 for the skin index, when coupled with COI compliance in the mid-CCT range, will ensure very satisfactory skin rendition and patient condition monitoring.

### 2.5. Mixed LED Lighting

We have investigated white-light mixtures using a range of seven coloured-LED sources [24]. The mixtures will use four LEDs at a time. Fewer than 4 lead to inferior colour properties, while more than 4 are assessed as too cumbersome for effective control. If a suitable LED combination can be established, then it becomes possible to achieve variations in the CCT of the mixture simply by varying the proportions of each constituent in the mixture. The benefit of such an approach is the simplification of the design of HCL for clinical settings.

We are not alone in making proposals for the implementation of colour-mixed LED lighting. Chiang and Chien [25], for example, have devised a “pentachromatic R/G/B/A/CW platform suitable for clinic use.” They propose an algorithm that permits their multispectral LED cluster to produce CCTs in the range 2800 K to 8000 K together with a high Colour Quality Scale (CQS > 85 points)—see [26].

One of the concerns with the use of LED mixtures is whether the mixture is capable of maintaining its target settings when subjected to the rigours of field use. Llenas and Carreras [27] have demonstrated that the problems that result from LED aging and temperature variations can be effectively eliminated in practice by the use of suitable feedback systems.

### 2.6. Optimization Tool

We have designed an optimization tool to find spectra that best meet the performance requirements for COI, colour rendition, and LER. It is based on a Matlab® program that derives the best 4-band mixtures of LEDs from a total set of seven available narrow-band LED spectra. Optimizations are performed by use of a DE (differential evolution) type of algorithm [28, 29] which has been demonstrated to be effective in several other spectral design settings [30, 31].

The search for an optimal SPD starts with a population of *P* randomly created candidate-solution vectors, each of which represents a possible combination of LED spectra. The value of *P* is kept constant during the optimization process. The candidate vectors undergo mutation, crossover, evaluation, and selection over a number of generations *G*. Both the population size *P* and the number of generations *G* depend on the problem to be optimised. In practice, it has been found that *P* must be >4 to provide sufficient mutually different solution vectors for the algorithm to function properly. Note that *G* is a user-selectable quantity, and larger values for *G* inevitably lead to longer execution times for the program.

Our algorithm also includes selections for two further DE settings: *F* is a mutation weight (between 0 and 2) which influences the magnitudes of mutations in the evolution process and CR (from 0 to 1) is a crossover constant ensuring that offspring solution vectors always differ from their parents. A sorting process ensures that only the fitter offspring vectors are moved to the succeeding generation.

We experimented with different settings for the DE parameters *P*, *F*, and CR, and found that the values of these parameters had only a minor influence on the optimization results. Our experience was that the process of choosing the control variables to achieve good optimization was straightforward. After some experimentation, we set *P*=10 and *F* and CR to the values suggested in [29], i.e., *F* = 0.5 and CR = 0.1.

Every DE process has to be controlled by a fitness function that determines how closely the members of a given generation have approached the required objective(s). In this case, the fitness function was set equal to the COI (equation (4)), and the set objective was that *f*_fit_ be minimised. The solution vector (in this case, SPD) having the lowest *f*_fit_, as well as CCT within range (i.e., 3300 ≤ CCT ≤ 5300 K), at the end of the final generation is regarded as the optimum:

(4) $$\begin{array}{c} f_{\text{fit}}=\text{COI}. \end{array}$$

For the purposes of this project, we performed over 140 independent optimization runs. We kept *P*=10 throughout and *G* of different durations (10, 20, 50, 100, and 200). Each value of *G* was applied in at least five separate optimization runs. Of the total number of runs, *G* of 10 was applied 50 times and *G* of 200 was used 65 times.

### 2.7. Selection of LED Spectra

We had access to the SPDs of seven coloured LEDs in the Lumileds LUXEON range [24]. For the first 75 optimization runs, the program was set to make a random selection of any 4 LEDs (from the 7 available) to form each of the members of the initial population for that run. In reviewing all 75 runs, it became evident that there were 4 specific LED spectra that consistently gave a range of “good” results as judged on the basis of colour rendition and LER performance as well as COI and CCT. These four LED spectra then became the focus of the next 65 optimization runs, from which a final total of 33 SPDs were selected as having potentially useful properties.

Since we wish to make a practical proposal for actual clinical lighting, we have subsequently made a selection of 8 mixtures (from the set of 33) to serve as basis for our recommendations.

## 3. Results and Discussion

Our selection of the four final LED colours (with peak wavelengths in brackets) was royal blue (450 nm), green (525 nm), amber (590 nm), and red (640 nm), and the results shown in Table 2 were obtained using these colours for optimum mixtures at specific CCTs. Note that the blue constituent of our mixture has its peak at 450 nm that is within the range for the stimulation of the alerting response of the circadian system [5].

### 3.1. Optimizing for COI

The CCTs were selected on the basis of (a) the acceptable range of CCT to provide good COI and (b) the ability to provide a CCT range that will support the circadian response. The selection of mixtures for CCTs outside of the preferred range for COI is covered in a later subsection.

The 33 optimised mixtures that conform to COI requirements are summarized in Table 2 which shows the various colorimetric and operational parameters for each mixture, sorted in order of increasing CCT. Note that several additional parameters have been listed in the table to provide more detailed information on the colour properties of each SPD.

By definition, the CRI value *R*_a_ [13] is based on the average colour difference for eight moderate-chroma colours, evenly spread around the hue circle. The CIE has also defined a supplementary set of six colours to provide more detail where necessary. Among these are the saturated red sample (index *R*_9_) and Caucasian skin colour analogue (index *R*_13_), and we have included these two indices along with *R*_a_.

In a similar way, the colour fidelity *R*_f_ is defined as an average colour difference for 99 different colours. Indices for specific colours can be extracted from the data available for the calculation of *R*_f_. Here, we have included *R*_fskin_ which the IESNA [14] defines as the average of two indices (for sample numbers 15 and 18) representing light and dark skin tones. In addition, we have extracted the index (*R*_fmin_) and its sample number (*i*_min_) for the sample having the lowest index value for a particular SPD since this indicates the worst-rendered colour under the specific source.

The eight SPDs in our final recommendation were selected for their superior properties in terms of colour performance and LER. To assist in the selection, we formulated a simple Overall Index (OI) as a general figure of merit, as defined in equation (5). Each of the selections was made on the basis of the highest OI value when compared with others fully meeting the COI criteria in the immediate CCT vicinity:

(5) $$\begin{array}{c} \text{OI}=R_{\text{a}}+R_{9}+R_{13}+R_{\text{f}}+R_{\text{fskin}}+R_{\text{fmin}}+\left(\frac{\text{LER}}{5}\right). \end{array}$$

**Figure 1:**
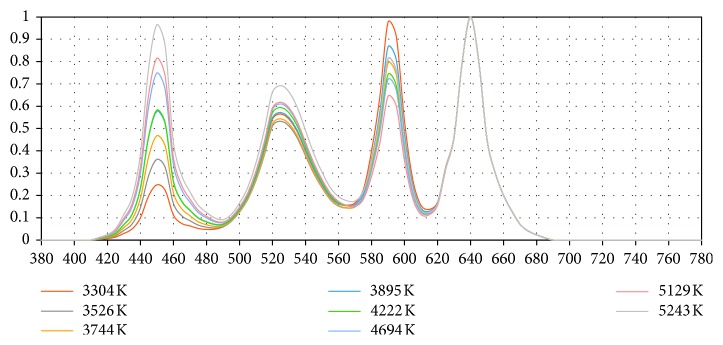
SPDs of the eight recommended LED mixtures.

Focussing now on the recommended eight SPDs, Figure 1 shows their SPDs on a single set of axes. All eight have been normalized to the red (640 nm) peak which all share as their common maximum.

In practice, suitable controls will need to be implemented to adjust the relative contributions to the mixture by each of the four LED colours. The controls should also be designed to enable dimming so as to permit the setting of different light levels at any selected CCT, thereby enhancing the HCL effects at appropriate times of the day.

**Figure 2:**
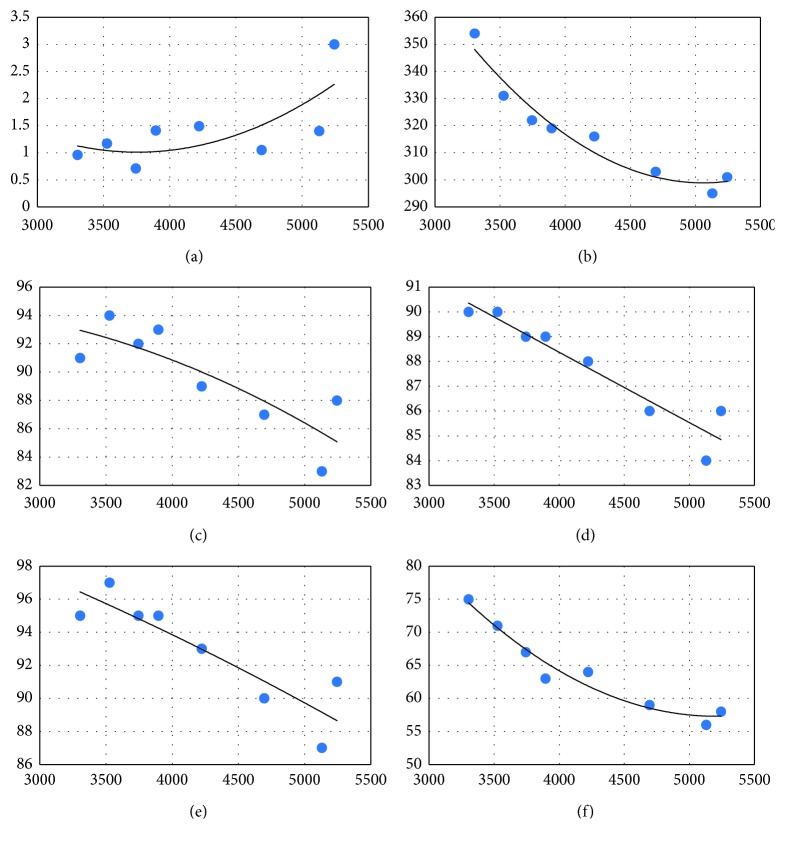
Relationship of source parameters to correlated colour temperature (CCT). Dependence of (a) COI, (b) LER, (c) *R*_a_, (d) *R*_f_, (e) *R*_13_, and (f) *R*_fmin_.

It is evident from Table 2 that the LER and all significant colour parameters are dependent on the CCT.  Figure 2 indicates the variations in a selection of these parameters for the recommended range of CCT settings. The curves shown are best-fit polynomials (order 2).

The COI (for which lower values are preferable) showed an upward (i.e., deteriorating) trend with CCT, and a maximum COI result of 3.0 for a CCT just below 5300 K. All the other depicted parameters also showed a deteriorating (now downward) trend. The LER was 354 lm/W at 3300 K and dropped about 17% to the region of 300 lm/W above 4500 K. The colour fidelity metrics, *R*_a_ and *R*_f_, both dropped from the low-90s region to approximately the mid-80s. The skin fidelity metric *R*_13_ fell from around 95 to about 89; and *R*_fmin_ (an indicator of the worst-case fidelity) fell from 75 to roughly 57. Note that *R*_fskin_ was ≥91 throughout and, together with the high *R*_13_ values, indicates that skin colour rendition will be good at all CCT settings.

Taken together, these results indicate that operation at 3300 K gives the best economy as well as the best COI and best general colour fidelity properties. It should be noted, however, that the above properties are, in fact, acceptable for the full range of CCTs depicted and there are likely to be situations when higher-CCT operation may be desired, e.g., to satisfy patient preferences, or for HCL purposes which call for lower CCT at night and higher CCT in the morning.

### 3.2. Optimizing for HCL

HCL (human-centric lighting) objectives are not restricted to the CCT range desired for COI, and we note that it is possible in principle to set the CCT of the mixture to values that fall outside the recommended range for optimum cyanosis observation. For example, it may be desirable in a hospital ward to set both CCT and lumen output to low values to promote a restful evening environment or to high values for a more stimulating morning and daytime environment.

A further optimization experiment was therefore conducted to find acceptable mixtures to achieve such variations without changing the constituent wavelengths in the mixture. The method was an adaptation of our earlier work [32] using differential evolution (DE) with a fitness function designed for optimization in the CIE 13.3 domain.

Table 3 gives a selection of the results that can be achieved and illustrates the feasibility of the approach. It shows that although outside the acceptable range of CCT for COI purposes, very good results are obtainable for *R*_a_ and *R*_f_, as well as for the skin colour indices *R*_13_ and *R*_fskin_. The chief drawback of the high-range CCTs is their rather poor *R*_fmin_ performance.

Note that 7500 K is higher than normally recommended for interior lighting as it represents the stronger blue light component of the sky during daylight, but it may be used when appropriate for an enhanced HCL stimulation response.

## 4. Conclusions

We have explained the importance of lighting from the perspective of human health and have shown how the human circadian system can best be stimulated for good health effects. We have also explained the value of cyanosis as a general indicator of health in the clinical setting and have shown how good spectral design of the lighting is best able to enhance cyanosis observation.

We have developed an optimization tool in Matlab® which invokes the Cyanosis Observation Index (COI) to provide the facility to optimize the lighting and so enhance the caregiver's ability to observe cyanosis. The recommended range of CCT for effective cyanosis observation is 3300–5300 K, and we have made recommendations for eight different mixtures of four LED spectra that give good values for the COI (i.e., <3.3) over this range.

We have utilized a previously developed CRI optimization tool which has enabled us also to recommend five further LED mixtures (using the same 4 LEDs as before) for CCTs which are below 3300 K or above 5300 K. These will allow the clinician to provide a wider range of different CCTs (when cyanosis observation is not continuously required) to help harness the benefits of human-centric lighting (HCL).

It is noteworthy that all the recommended mixtures provide excellent LER (285 ≤ LER ≤ 354 lm/W), together with good colour rendition in terms of the CRI (*R*_a_ and *R*_13_) of the CIE as well as the colour fidelity index (*R*_f_) and colour skin index (*R*_fskin_) of the IESNA.

We note that the blue LED in our recommended mixtures has a peak wavelength of 450 nm and a bandwidth (FWHM) of 18 nm (441–459 nm). It is therefore capable of providing significant energy for effective HCL activity within the recommended (446–477 nm) band—particularly when operating in the higher-CCT mixtures. Conversely, for low-CCT mixtures (in which the blue content is significantly cut), there will be low activation, as required for the creation of a restful night-time ambience.

There are numerous other ways in which lighting is able to enhance the patient experience and health outcomes [33], and we have not attempted to include them all in this study. We have focussed here on the more general areas of patient lighting as they may apply in both day-stay and overnight hospital wards or clinics.

## Figures and Tables

**Table 1:** Recommended source colour properties in codes and standards.

Country or region	Australia and New Zealand [10]	United Kingdom [15] and European Union [16]	United States [17]
Colour appearance (CCT)	3300 K ≤ CCT ≤ 5300 K (all areas)	No general recommendation	2700 K night lighting. No other general recommendation

Colour rendering index—general and ward areas	*R*_a_ ≥ 80	*R*_a_ ≥ 80	No general recommendation

Colour rendering index—examination and treatment areas	*R*_a_ ≥ 85 (e.g., dermatology)	*R*_a_ ≥ 90 (all examination areas)	*R*_a_ ≥ 80 for exam areas *R*_a_ > 90 for surgery

Special colour requirements	Cyanosis observation: COI ≤ 3.3	Examination rooms (general lighting): 4000 K ≤ CCT ≤ 5000 K Colour inspection (labs and pharmacies): 6000 K ≤ CCT ≤ 6500 K Consider the appropriate special colour rendering index *R*_i_ for accurate rendition of coloured objects or human skin	Surgery and examination rooms: 4000 K ≤ CCT ≤ 5000 K

Human-centric lighting (reinforcing circadian entrainment)	Not included	Lighting may have a significant influence on human circadian rhythms	Lighting practitioners to be aware of principles of the circadian cycle

*Note.* This table represents a distillation, by the authors, of detailed and sometimes complex requirements or recommendations. Please refer to the original documents for full details and explanations.

**Table 2:** 33 optimised mixtures of the four selected LED colours.

SPD no.	CCT (K)	COI	CIE CRI results				IES TM-30-15 results					Surface colour giving *R*_fmin_	OI
			*R*_a_	*R*_9_	*R*_13_	LER (lm/w)	*R*_f_	*R*_g_	*R*_fskin_	*R*_fmin_	*i*_min_		
1	3304	0.96	**91**	**49**	**95**	354	**90**	99	**91**	75	40	Dark greenish-grey (F)	562
2	3311	1.49	**90**	**40**	**92**	362	**89**	95	**89**	74	42	Olive-green (F)	546
3	3365	1.22	**93**	**61**	**98**	339	**89**	103	**91**	67	20	Orange-brown (F)	567
4	3381	0.88	**92**	**59**	**97**	344	**90**	102	**92**	73	20	Dark orange (F)	572
5	3401	2.2	**94**	**88**	**99**	339	**91**	103	**96**	72	42	Olive-green (F)	608
6	3444	0.96	**93**	**68**	**98**	344	**90**	102	**93**	74	42	Olive-green (F)	585
7	3462	0.85	**93**	**69**	**99**	336	**90**	104	**93**	68	20	Orange-brown (F)	579
8	3488	1.29	**94**	**81**	**97**	326	**89**	106	**93**	62	20	Orange-brown (F)	581
9	3526	1.17	**94**	**91**	**97**	331	**90**	105	**96**	71	42	Olive-green (F)	605
10	3607	1.49	**92**	**66**	**97**	182	**90**	102	**93**	74	42	Olive-green (F)	548
11	3608	1.27	**94**	**85**	**96**	324	**89**	107	**94**	62	20	Orange-brown (F)	585
12	3716	0.86	**92**	**98**	**94**	317	**89**	108	**96**	63	20	Dark orange (F)	595
13	3744	0.71	**92**	**96**	**95**	322	**89**	107	**97**	67	42	Olive-green (F)	600
14	3744	3.11	**93**	**81**	**95**	317	**88**	108	**92**	55	20	Dark orange (F)	567
15	3753	1.17	**91**	**84**	**90**	310	**88**	110	**97**	62	20	Orange-brown (F)	574
16	3880	1.24	**93**	**91**	**96**	323	**89**	107	**96**	65	20	Orange-brown (F)	595
17	3895	1.41	**93**	**94**	**95**	319	**89**	108	**96**	63	20	Orange-brown (F)	594
18	4060	2.17	**93**	**80**	**99**	329	**89**	105	**95**	68	20	Dark orange (F)	590
19	4199	2.49	**80**	**24**	**79**	286	**83**	115	**92**	55	42	Olive-green (F)	470
20	4222	1.49	**89**	**79**	**93**	316	**88**	108	**98**	64	42	Olive-green (F)	574
21	4277	0.92	**90**	**79**	**91**	309	**87**	109	**98**	62	42	Olive-green (F)	569
22	4308	3.33	**92**	**97**	**93**	193	**88**	108	**96**	59	20	Orange-brown (F)	564
23	4431	0.83	**88**	**69**	**90**	306	**87**	110	**97**	61	42	Olive-green (F)	553
24	4669	1.5	**82**	**32**	**81**	286	**83**	114	**93**	54	42	Olive-green (F)	482
25	4694	1.05	**87**	**61**	**90**	303	**86**	110	**96**	59	42	Olive-green (F)	540
26	4944	1.33	**83**	**33**	**82**	287	**83**	114	**93**	54	42	Olive-green (F)	485
27	5111	0.82	**83**	**37**	**84**	291	**84**	113	**94**	54	42	Olive-green (F)	494
28	5129	1.4	**83**	**38**	**87**	295	**84**	112	**94**	56	42	Olive-green (F)	501
29	5148	2.44	**83**	**44**	**83**	287	**83**	113	**94**	53	42	Olive-green (F)	497
30	5196	1.44	**77**	**4**	**76**	277	**81**	116	**90**	49	42	Olive-green (F)	432
31	5211	0.77	**82**	**31**	**84**	290	**83**	114	**93**	54	42	Olive-green (F)	485
32	5243	3	**88**	**75**	**91**	301	**86**	110	**97**	58	42	Olive-green (F)	555
33	5331	3.37	**88**	**77**	**91**	301	**86**	110	**97**	57	42	Olive-green (F)	556

Final recommendation: SPD nos. 1, 9, 13, 17, 20, 25, 28, and 32. Note that SPDs 22 and 33 are slightly outside the COI recommendations. Colour descriptions are our own interpretations of TM-30-15 test colours; type *F* are print colours.

**Table 3:** Optimised mixtures for low and high CCTs.

CCT (K)	CIE CRI results			LER (lm/w)	IES TM-30-15 results					Surface colour giving *R*_fmin_	OI
	*R*_a_	*R*_9_	*R*_13_		*R*_f_	*R*_g_	*R*_fskin_	*R*_fmin_	*i*_min_		
Low											
2759	**95**	87	**98**	332	**89**	100	**94**	71	25	Yellowish-brown (A)	600
3051	**94**	74	**100**	340	**90**	101	**94**	75	42	Olive-green (F)	595
High											
5734	**90**	95	**98**	309	**86**	107	**97**	57	75	Dark blue (F)	585
6563	**89**	95	**100**	305	**86**	106	**97**	53	75	Dark blue (F)	581
7539	**86**	71	**90**	285	**83**	110	**96**	50	75	Dark blue (F)	533

Colour descriptions are our own interpretations of the TM-30-15 test colours; type A are from nature; type F are print colours.

## Data Availability

The data used to support the findings of this study are available from the corresponding author upon request.

## References

[1] SCHEER (Scientific Committee on Health, Environmental and Emerging Risks) (2018). *Opinion on Potential Risks to Human Health of Light Emitting Diodes (LEDs)*.

[2] Genova C. Normal responses to non-visual effects of light retained by blind humans lacking rods and cones. https://www.medicalnewstoday.com/releases/91836.php.

[3] van Bommel W., van den Beld G. (2004). Lighting for work: a review of visual and biological effects. *Lighting Research & Technology*.

[4] Pechacek C. S., Andersen M., Lockley S. W. (2008). Preliminary method for prospective analysis of the circadian efficacy of (day) light with applications to healthcare architecture. *LEUKOS*.

[5] Andersen M., Mardaljevic J., Lockley S. W. (2012). A framework for predicting the non-visual effects of daylight, Part 1 photobiology-based model. *Lighting Research & Technology*.

[6] Meadows C. Setting expectations on human-centric lighting. https://www.ledsmagazine.com/articles/2017/12/setting-expectations-on-human-centric-lighting.html.

[7] Midolo N. A., Sergeyeva L. (2007). Lighting for clinical observation of cyanosis. *The Australian Hospital Engineer*.

[8] Changizi M., Rio K. (2010). Harnessing color vision for visual oximetry in central cyanosis. *Medical Hypotheses*.

[9] Snider H. L., Walker H. K., Hall W. D., Hurst J. W. (1990). Cyanosis. *Clinical Methods: The History, Physical, and Laboratory Examinations*.

[10] AS/NZS 1680.2.5:2018, Australian/New Zealand Standard: Interior and Workplace Lighting, Part 2.5: Hospital and Medical Tasks, Section 7.3 Cyanosis Observation Lighting, and Appendix G: Method for Determination of Cyanosis Observation Index of a Light Source

[11] Commission Internationale de l’Eclairage (2003). *Colorimetry*.

[12] Commission Internationale de l’Eclairage (2017). *International Lighting Vocabulary*.

[13] Commission Internationale de l’Eclairage (1995). *Method of Measuring and Specifying Colour Rendering Properties of Light Sources*.

[14] Illuminating Engineering Society of North America (2015). *IES Method for Evaluating Light Source Color Rendition*.

[15] The Society of Light and Lighting (2008). *Lighting Guide 2: Hospitals and Health Care Buildings*.

[16] BSI Standards Publication (2011). *BS EN 12464-1:2011: Light and Lighting—Lighting of Work Places: Part 1: Indoor Work Places*.

[17] ANSI/IES (2016). *RP-29-16: Lighting for Hospitals and Healthcare Facilities*.

[18] Alzubaidi S., Soori P. K. (2012). Energy efficient lighting system design for hospitals diagnostic and treatment room—a case study. *Journal of Light & Visual Environment*.

[19] Leccese F., Montagnani C., Iaia S., Rocca M., Salvadori G. (2016). Quality of lighting in hospital environments: a wide survey through in situ measurements. *Journal of Light & Visual Environment*.

[20] Thorn Lighting Ltd. *Thorn Applications and Techniques: Healthcare*.

[21] Mehrotra S., Basukala S., Devarakonda S. (2015). Effective lighting design standards impacting patient care: a systems approach. *Journal of Biosciences and Medicines*.

[22] Bartczak P., Gebejes A., Falt P., Hauta-Kasari M. An LED-based tunable illumination for diverse medical applications.

[23] Litorja M., Ecker B. Use of a spectrally-tunable source to explore improvement in chromatic contrast for illumination of tissues.

[24] Lumileds Lighting (2006). LUXEON®, K2 emitter. *Technical Datasheet DS51*.

[25] Chiang S. B., Chien K. The optimization of color-mixed LED lighting.

[26] Davis W., Ohno Y. (2010). Color quality scale. *Optical Engineering*.

[27] Llenas A., Carreras J. (2019). Arbitrary spectral matching using multi-LED lighting systems. *Optical Engineering*.

[28] Storn R., Price K. (1995). Differential evolution—a simple and efficient adaptive scheme for global optimization over continuous spaces.

[29] Storn R., Price K. (1997). Differential evolution—a simple and efficient heuristic for global optimization over continuous space. *Journal of Global Optimization*.

[30] Soltic S., Chalmers A. (2012). Differential evolution for the optimisation of multi-band white LED light sources. *Lighting Research & Technology*.

[31] Chalmers A. N., Soltic S. (2012). Light source optimization: spectral design and simulation of four-band white-light sources. *Optical Engineering*.

[32] Soltic S., Chalmers A. N. Differential evolution and its application to intelligent spectral design.

[33] Schlangen L. J. M. (2010). *The Role of Lighting in Promoting Well-Being and Recovery Within Healthcare*.

